# First Description of Ceftazidime/Avibactam Resistance in a ST13 KPC-70-Producing *Klebsiella pneumoniae* Strain from Portugal

**DOI:** 10.3390/antibiotics11020167

**Published:** 2022-01-27

**Authors:** Gabriel Mendes, João F. Ramalho, Ana Bruschy-Fonseca, Luís Lito, Aida Duarte, José Melo-Cristino, Cátia Caneiras

**Affiliations:** 1 Microbiology Research Laboratory on Environmental Health (EnviHealthMicro Lab), Institute of Environmental Health (ISAMB), Faculty of Medicine, Universidade de Lisboa (ULisboa), 1649-028 Lisboa, Portugal; gabriel-mendes@campus.ul.pt (G.M.); jfrancisko.ramalho@gmail.com (J.F.R.); 2 Microbiology Laboratory, Clinical Pathology Department, Centro Hospitalar Universitário Lisboa Norte, 1649-035 Lisboa, Portugal; ana.bruschy@chln.min-saude.pt (A.B.-F.); lmlito@chln.min-saude.pt (L.L.); melo_cristino@medicina.ulisboa.pt (J.M.-C.); 3 Faculty of Pharmacy, Universidade de Lisboa (ULisboa), 1649-033 Lisboa, Portugal; aduarte@ff.ulisboa.pt; 4 Egas Moniz Interdisciplinary Research Center, Egas Moniz University Institute, 2829-511 Monte da Caparica, Portugal; 5 Institute of Microbiology, Faculty of Medicine, Universidade de Lisboa (ULisboa), 1649-028 Lisboa, Portugal; 6 Institute of Preventive Medicine and Public Health, Faculty of Medicine, Universidade de Lisboa (ULisboa), 1649-028 Lisboa, Portugal

**Keywords:** KPC-70, *Klebsiella pneumoniae*, ceftazidime/avibactam resistance, KPC-3, ST13, KPC-variant, hypermucoviscosity, hypervirulence, Portugal

## Abstract

The combination of ceftazidime/avibactam (CZA) is a novel β-lactam/β-lactamase inhibitor with activity against *Klebsiella pneumoniae* carbapenemase (KPC)-producing Enterobacterales. Emerging cases caused by CZA-resistant strains that produce variants of KPC genes have already been reported worldwide. However, to the best of our knowledge, no CZA-resistant strains were reported in Portugal. In September 2019, a *K. pneumoniae* CZA-resistant strain was collected from ascitic fluid at a surgery ward of a tertiary University Hospital Center in Lisboa, Portugal. The strain was resistant to ceftazidime/avibactam, as well as to ceftazidime, cefoxitin, gentamicin, amoxicillin/clavulanic acid, and ertapenem, being susceptible to imipenem and tigecycline. A hypermucoviscosity phenotype was confirmed by string test. Whole-genome sequencing (WGS) analysis revealed the presence of an ST13 KPC70-producing *K. pneumoniae*, a KPC-3 variant, differing in two amino-acid substitutions (D179Y and T263A). The D179Y mutation in the KPC Ω-loop region is the most common amino-acid substitution in KPC-2 and KPC-3, further leading to CZA resistance. The second mutation causes a KPC-70 variant in which threonine replaces alanine (T263A). The CZA-resistant strain showed the capsular locus KL3 and antigen locus O1v2. Other important virulence factors were identified: fimbrial adhesins type 1 and type 3, as well as the cluster of iron uptake systems aerobactin, enterobactin, salmochelin, and yersiniabactin included in integrative conjugative element 10 (ICE*Kp10*) with the genotoxin colibactin cluster. Herein, we report the molecular characterization of the first hypervirulent CZA-resistant ST13 KPC-70-producing *K. pneumoniae* strain in Portugal. The emergence of CZA-resistant strains might pose a serious threat to public health and suggests an urgent need for enhanced clinical awareness and epidemiologic surveillance.

## 1. Introduction

*Klebsiella pneumoniae* belongs to the order Enterobacterales and is one of the most common nosocomial pathogens worldwide, representing a serious threat to clinical and public health [1]. Carbapenemase-producing Enterobacterales, such as *K. pneumoniae* producing carbapenemase (KPC-*Kp*), are resistant to almost all β-lactam inhibitors, including carbapenem, leading to scarce therapeutic options [1]. In Portugal, an increasing trend of carbapenem resistance has been reported by the European Center for Disease Prevention and Control (ECDC) [2] with an overlapping of multidrug resistance and virulence determinants in KPC-3 *K. pneumoniae* clinical strains that impose an additional challenge in the treatment of infections caused by this pathogen [3]. Furthermore, despite the improvements in recent years, Portugal has been identified as one of the countries in Europe with the highest rate of hospital infections [4] and with an increasing resistance tendency to the latest therapeutic lines available [5].

A recently developed drug, ceftazidime/avibactam (CZA), combines a third-generation cephalosporin (ceftazidime) and a non-β-lactam β-lactamase inhibitor (avibactam) that inactivates most Amber class A (including KPC), class C, and class D β-lactamases, but it is not effective against class B (metallo-β-lactamase-producing strains) [6]. CZA is approved for use in Europe since 2016 for the treatment of complicated intra-abdominal infections (cIAI), complicated urinary tract infections (cUTI) including pyelonephritis, hospital-acquired pneumonia (HAP) including ventilator-associated pneumonia (VAP), and infections due to aerobic Gram-negative organisms in patients with limited treatment options [7].

A high rate of clinical success and survival with ceftazidime/avibactam treatment regimen has been demonstrated in patients with infections caused by *K. pneumoniae* carbapenemase-producing Enterobacterales [8,9,10], and it has become an important first-line option [11]. However, despite the limited use of CZA worldwide, emerging ceftazidime/avibactam-resistant strains that produce variants of KPC genes have already been reported [12,13,14,15,16,17] and may represent a serious cause of concern [18], despite not being reported to date in Portugal. Thus, this article aims to describe the genomic molecular characterization of a CZA-resistant *K. pneumoniae* strain that was identified at a Tertiary University Hospital Center in Lisboa, leading, to the best of our knowledge, to the first description of a CZA-resistant strain in Portugal.

## 2. Results

The clinical strain was recovered from a 65 year old patient admitted at a Tertiary University Hospital Center in Lisboa, Portugal, in September 2019. The ceftazidime/avibactam-resistant *K. pneumoniae* was obtained from the biological product ascitic fluid and from a surgery hospital ward. After its identification, the antimicrobial susceptibility profiling analysis indicated that the strain was resistant to ceftazidime/avibactam, without an inhibition zone, as well as to ceftazidime, cefoxitin, gentamicin, amoxicillin/clavulanic acid, and ertapenem, being susceptible to imipenem and tigecycline and susceptible with increased exposure to ciprofloxacin, cefotaxime, aztreonam, and doripenem (Table 1). The hypermucoviscosity phenotype was confirmed by the string test.

MLST analyses of internal fragments of seven housekeeping genes revealed that the *K. pneumoniae* clinical strain belonged to sequence type 13 (ST13). In accordance with Table 2, and regarding the resistance determinants, we initially identified a carbapenemase-coding gene (*bla*_KPC_) by PCR screening. After WGS and using the BLAST and the Clustal Omega programs, we confirmed that the *bla*_KPC-70_ gene was a variant of the *bla*_KPC-3_ gene with two single-amino-acid variants: D179Y and T263A. Beyond the carbapenemase, three genes coding for narrow-spectrumβ-lactamases were found: *bla*_TEM-1_, *bla*_SHV-1_, and *bla*_OXA-9_. The *aac(6′)-Ib′*, *aadA*, *aph(3″)-Ib*, and *aph(6)-Id* genes producing aminoglycoside-modifying enzymes were also detected. Trimethoprim–sulfamethoxazole resistance was encoded by *dfrA14* and *sul2* genes, respectively. The *fosA* gene, which codes for fosfomycin resistance, was also identified. One mutation in the chromosomal *gyrA* locus was identified at the *gyrA*-87N gene, while a homology of only 70% was found in the *OmpK35* porin gene. Using the criterion of >95% nucleotide identity for large plasmids and >80% for Col-like plasmids, as well as >96% coverage with the reference replicon sequences, we found two plasmid replicon types IncFIA (pBK30683) and IncFII (pBK30683) and ColRNAI. These plasmids can contribute to the evolution of the clone by hosting the *bla*_KPC_ variants, as well as to rearrangement (gain or loss of additional resistance determinants) and fusion with coresident plasmids [19]. The genetic context of *bla*_KPC-70_ was found totally in the same contig as part of the Tn*4401d* isoform, characterized by a 68 bp deletion between *istB* and *bla*_KPC_ genes when compared with the Tn*4401b* isoform, which is a Tn*3*-based transposon involved in *bla*_KPC_ gene dissemination [20]. Moreover, regarding the virulence genes in *K. pneumoniae*, the antigen O encoded on *rfb* locus type O1v2 and the polysaccharide capsule are encoded in K-loci (KL3), while the *rcsA* and *rcsB* genes responsible for regulation of capsule synthesis were also found, but not the *rmpA* gene regulator of mucoid phenotype A. Others important virulence factors identified were fimbria adhesins type 1 (*fimA*–*fimK* genes) and type 3 (*mrkA*–*mrkJ* genes), as well as the iron uptake systems *kfu*, enterobactin cluster (*entA*–*fes*), aerobactin (*iutA*), salmochelin (*iroN* and *iroE*), and yersiniabactin cluster (*fyuA*–*ybtX*), which was included in integrative conjugative element 10 (ICE*Kp10*) with the genotoxin colibactin cluster (12 out of 18 genes). The hypermucoviscosity phenotype was confirmed.

## 3. Discussion

Ceftazidime/avibactam is a recent therapeutic option for treating serious infections caused by carbapenem-resistant *K. pneumoniae* (CRK) [9]. However, increased reports of CZA resistance in CRK are worrisome [16,17,21]. In Portugal, no reports of CZA resistance were found to date. In this study, we report a *K. pneumoniae* KPC-70 CZA^R^ strain that produces a *bla*_KPC-70_ gene, a *bla*_KPC-3_ variant with two single-amino-acid variants (D179Y and T263A).

The first mutation in the KPC Ω-loop region (D179Y) is the most common amino-acid substitution of KPC in KPC-2 [21] and KPC-3 [22,23]. It has been previously shown that mutations occurring at the 164–179 site result in flexibility of the Ω-loop region, which decreases the binding of avibactam and ceftazidime hydrolysis activity, leading to CZA resistance [24]. The KPC-70 variant, recently described in Italy [12], presents a D179Y mutation in the KPC Ω-loop region and a second mutation in which threonine replaces alanine (T263A).

Multilocus sequence typing (MLST) revealed that the *K. pneumoniae* clinical strain belonged to sequence type 13 (ST13). A previous study detected seven KPC-3-producers strains belonging to ST13 in Portugal [25] despite the genomic population structure being apparently dominated by ST147 [25,26]. In the remainder of the European continent, most ST13 *K. pneumoniae* strains were identified as OXA-48-like producers [27,28,29]. In contrast, KPC-producing clones were recently reported among South American public hospitals [30,31,32]. Nevertheless, none of these studies found CZA-resistant strains. In fact, although there has been an evident increase in CZA-resistant *K. pneumoniae* reports in the last few years [12,15,18,21] none of them exhibited an ST13 allelic profile, which highlights the importance of genomic surveillance of this ST13 clone.

Concerning virulence factors that have been characterized for *K. pneumoniae*, there are different types according to infection source, as well as *K. pneumoniae* ST strain. However, there are four major classes of virulence factors: capsule, including the production of hypermucoviscosity, lipopolysaccharide (LPS), siderophores, and fimbriae [33]. The iron acquisition systems are essential in *K. pneumoniae* infections, generally to control host defenses by inducing the dissemination through chelation of host cellular iron and regulating the production of multiple bacterial virulence factors [34]. Four principal siderophore systems were found in our strain. Firstly, enterobactin (*ent*) is common to all *K. pneumoniae* and conserved in the chromosome as core gene. Secondly, yersiniabactin is the most common virulence factor associated with human *K. pneumoniae* infections and enhances bacterial survival in the host [33,34]. Usually, this siderophore is included in an integrative and conjugative element (ICE) ICE*Kp10*, which in our study was harboring yersiniabactin (*ybt17*) and colibactin (*clb3*), which have a genotoxin effect on host cells by crosslinking DNA and disrupting host immune response [35]. Most ICEs are extremely prevalent in hypervirulent *K. pneumoniae* lineages. In particular, ICE*Kp10* is the most common type found in ST23 and ST258 [36]. The remaining siderophore gene clusters found encode for the biosynthesis of aerobactin and salmochelin. They are associated with invasive disease and are common amongst hypervirulent *K. pneumoniae* clones that cause severe community-associated infections such as liver abscesses and pneumonia. These siderophores were considered as the primary virulence determinant among iron acquisition systems in hypervirulent *K. pneumoniae* species [37], representing key genes for high virulence scores [38]. The other iron uptake system, *kfu*, is associated with invasive infections, capsule formation, and hypermucoviscosity [33]. Our CZA-resistant strain also presented a hypermucoviscosity (HMV) phenotype and genes coding for biofilm production, which are two key factors for colonization and persistence in the host. Notably, infection with biofilm-producing KPC-*Kp* strains was an independent predictor of mortality [39]. Worryingly, our strain revealed the presence of the type 3 fimbriae *mrkA–mrkJ* cluster, including the type 3 fimbriae-related coding genes (*mrkA* and *mrkD*) and regulation gene (*mrkH*). The biofilm formation capacity of strains that carried the *mrkH* cassette was previously reported as significantly higher than other strains considering that the expression of *mrkA* in *K. pneumoniae* carrying the *mrkH* gene was significantly upregulated [40]. Indeed, the assessment of biofilm production may provide a key element in supporting the clinical management of high-risk patients with KPC-*Kp* infection [39] and a relevant emerging problem [39,40,41].

Additionally, the CZA-resistant strain reported herein produced a capsular type K3 (considered the usual cause of rhinoscleroma) [42], not previously associated with hvKp, since it is not amongst the most common capsule loci associated with hvKp—K1, K2, K5, and K57 [36]. Nevertheless, the string test confirmed the hypermucoviscosity phenotype of our strain; the PCR and WGS analyses were negative for *magA* gene (considered to be restricted to K1 strains) [36], as well as for *rmpA* and *rmpA2*. However, given that our strain was isolated from an ascitic fluid, the number of virulence factors found (including relevant siderophores as aerobactin and yersiniabactin), associated with resistance to last-line antibiotic ceftazidime/avibactam, it can be characterized as an hvKp strain. However, this is still a controversial concept. In the literature, hypermucoviscous and hypervirulent are often used synonymously, but studies found that the string test performed could be a predictor of hypervirulent strains, and that most hypermucoviscosity phenotypes likely contribute to the majority of hvKp strains [36]. The emergence of hvKp strains is a great cause for concern, as they successfully escape immune system mechanisms due to their high number of virulence factors. Because genes that encode for virulence or antibiotic resistance are often on mobile genetic elements such as plasmids, transposons, and ICE, it is not surprising that we are observing a convergence of virulence and antibiotic resistance. Despite this fact, the interplay between antimicrobial resistance and virulence still remains poorly understood worldwide, particularly in the convergence of hvKp and CZA-resistant strains.

Interestingly, it has been described that, during CZA administration, the MIC of meropenem (MEM) can decrease and some KPC-producing *K. pneumoniae* strains can result in CZA-resistant and MEM-susceptible strains [43,44]. Indeed, our CZA-resistant strain is MEM-susceptible considering the EUCAST category. However, this information should be interpreted with caution by the clinicians. Previous studies confirmed the difficulty of treating infections caused by KPC-*Kp*, which, under CZA-treatment, can rapidly evolve from CZA-susceptible to CZA-resistant via the emergence of KPC-3 variants. These KPC-3 variant-producing strains can be treated with MEM, but the colonization persistence of MEM-resistant KPC-3 producers can lead to treatment failure [19] with high mortality rates [18]. In fact, the role of carbapenems in treating such infections remains uncertain, because meropenem resistance is readily selected during passage experiments [43].

Ceftazidime/avibactam has definitely become an important first-line option due the limited therapeutic options available for KPC-*Kp* infections, which was recently recognized by Infectious Diseases Society of America (IDSA) guidelines that indicate CZA, meropenem/vaborbactam, and imipenem/cilastatin/relebactam as the preferred agents for KPC-*Kp* infections outside of the urinary tract [11]. Previous existing options, such as polymyxins, aminoglycosides, tigecycline, and carbapenems, were of limited use due to inferior efficacy, resistance rates, suboptimal doses, and high toxicity [45]. Therefore, it is imperative to encourage CZA use from an antimicrobial stewardship perspective in order to retain the activity against KPC-*Kp* strains. However, the data currently available highlight that an optimal therapeutic regimen for CZA or for patients infected by CZA-resistant strains remains lacking [18]. In Italy, a case of a 68 year old man with recurrent bacteremia caused by a KPC-*Kp* strain resistant to ceftazidime/avibactam and cefiderocol was reported. A KPC-3 variant enzyme (D179Y; KPC-31) was identified, which confers resistance to ceftazidime/avibactam and restores meropenem susceptibility. The patient that was successfully treated with meropenem/vaborbactam [46], which previously demonstrated potential to be an effective option as salvage therapy for ceftazidime/avibactam-resistant KPC-producing *K. pneumoniae* infections [47].

## 4. Materials and Methods

### 4.1. Bacterial Strain

The strain was recovered using standard clinical operating procedures. The identification was performed by microbiology laboratories using conventional methods or automated systems such as Vitek2^®^ (BioMérieux, Marcy, l’Étoile, France) or MicroScan (Snap-on, Kenosha, WI, USA). The strain was frozen in brain heart infusion (BHI) broth (VWR Prolabo, Lisboa, Portugal) with 15% glycerol at −80 °C. For analysis, the strain was grown using BHI broth (18 h, 37 °C) and later seeded in Mueller–Hinton agar (VWR Prolabo, Lisboa, Portugal).

### 4.2. Antimicrobial Susceptibility Testing and Hypermucoviscosity Phenotype

Antimicrobial susceptibility testing was performed using the standardized Kirby– Bauer disc diffusion technique, in accordance with the European Committee on Antimicrobial Susceptibility Testing (EUCAST). The detailed methodology is available at http://www.eucast.org/ast_of_bacteria/disk_diffusion_methodology/ (accessed on 17 December 2021). Detailed instructions for Mueller–Hinton agar medium (VWR Prolabo^®^, Lisboa, Portugal), including preparation and storage, are also available in the same EUCAST guidelines document. Quality control was carried out in accordance with EUCAST (version 11.0, 2021). Susceptibility was tested by a panel of antibiotics: amoxicillin/clavulanic acid (20/10 µg), cefoxitin (30 µg), cefotaxime (5 µg), ceftazidime (10 µg), imipenem (10 µg), gentamicin (10 µg), ciprofloxacin (5 µg), tigecycline (15 µg), aztreonam (30 µg), ertapenem (10 µg), doripenem (10 µg), meropenem (10 µg), and ceftazidime/avibactam (10/4 µg) (Biorad, Portugal). The strains were categorized as susceptible to standard dosing regimen (S), susceptible to increased exposure (I), and resistant (R) by applying the breakpoints in the phenotypic test results. A complementary Etest^®^ (BioMérieux, Marcy l’ Étoile, France) for ceftazidime/avibactam was also performed. The inhibition zones and MIC values were interpreted according to the EUCAST breakpoints (version 11.0, 2021) (available at https://eucast.org/clinical_breakpoints/ (accessed on 17 December 2021)).

The hypermucoviscosity phenotype is a known virulent factor which was determined using a simple method, the string test. A positive string test is defined as the formation of viscous strings of >5 mm in length when a loop is used to stretch the colony on an agar plate. Multidrug-resistant (MDR) bacteria were defined as those that acquired nonsusceptibility to at least one agent in three or more antimicrobial categories, in accordance with the United States Center for Disease Control and Prevention (CDC) and the European Center for Disease Prevention and Control (ECDC) consensual definition [48].

### 4.3. Resistance and Virulence Determinants

PCR-based screening was performed to identify carbapenemase genes with primers designed for *bla*_OXA-48_ (F: 5′–GGCTGTGTTTTTGGTGGCATC–3′; R: 5′–GCAGCCCTAAACCATCCGATG–3′), *bla*_KPC_ [49], *bla*_VIM_ [50], *bla*_NDM_ [51], and *bla*_GES_ [52]. The virulence factors were also assessed by PCR with specific primers for the fimbrial adhesins type 1 (*fimH*) [53], hemolysin (*khe*), iron uptake system (*kfu*) [54], siderophore (*enterobactin*, *entB*), and the hypermucoviscosity phenotype (*magA*) [55].

### 4.4. Molecular Methods

Polymerase chain reaction (PCR) was performed using the NZYTaq II 2× Green Master Mix (NZYTech, Lisboa, Portugal) following the manufacturer’s instructions. The PCR products were resolved in 1% agarose gel in 10× concentrated Tris–borate–EDTA (TBE buffer) (NZYTech, Lisboa, Portugal). Positive and negative controls were included in all PCR assays. The positive controls were used with strains containing the respective genes and previously sequenced. The purification of PCR amplification products was performed using the ExoCleanUp FAST (VWR Prolabo^®^, Lisboa, Portugal) kit, and they were sequenced at STABVida Portugal. Searches for nucleotide sequences were performed with the BLAST program, which is available at the National Center for Biotechnology Information website (http://www.ncbi.nim.nih.gov/ accessed on 17 December 2021). Multiple-sequence alignments were performed with the Clustal Omega program, which is available at https://cge.cbs.dtu.dk/services (accessed on 17 December 2021).

### 4.5. Multilocus Sequence Typing (MLST)

MLST was performed by analyses of internal fragments of seven housekeeping genes (*gapA*, *infB*, *mdh*, *pgi*, *phoE*, *rpoB*, and *tonB*), as previously described [56]; the sequencing was performed at STABVida Portugal and submitted to the MLST database for allele attribution. The *K. pneumoniae* database is available at the Pasteur MLST website (http://www.pasteur.fr/mlst/ (accessed on 17 December 2021)).

### 4.6. Whole-Genome Sequencing

The genomic DNA of the clinical strain was extracted from cultures grown overnight, in Mueller–Hinton agar, using the NZY Tissue gDNA Isolation kit (NZYTech, Lisboa, Portugal), as per the manufacturer’s recommendations. The sequencing was performed at STABVida Portugal. Sequencing libraries were prepared using the KAPA HyperPrep Library Preparation Kit (Roche, Switzerland) following the manufacturer’s recommended protocol and sequenced using an Illumina HiSeq Novaseq 6000 platform with paired-end reads (2 × 151 bp). The raw data quality control was performed using FASTQC v0.11.9, and the trimming and de novo assembly were performed using CLC Genomics Workbench 12.0.3 (QIAGEN, Aarhus, Denmark). All assemblies were carried out with automatic word size, a similarity fraction of 0.95, a length fraction of 0.95, and a minimum contig size of 500 bp.

### 4.7. Drug Resistance-Associated Genes, Virulence Genes, Capsular Types, and Plasmid Replicons

Antimicrobial resistance (AMR) K and O antigen loci were identified using Kleborate (https://github.com/katholt/Kleborate (accessed on 17 December 2021)), a *K. pneumoniae*-specific genomic typing tool, and virulence factors using the virulence factor database VFDB (http://www.mgc.ac.cn/VFs/ accessed on 17 December 2021). Plasmid analyses were identified using the PlasmidFinder database with the following cutoff values: minimum of 60% coverage and 95% identity (https://cge.cbs.dtu.dk/services/PlasmidFinder/ (accessed on 17 December 2021)).

### 4.8. Accession Number

The sequence was submitted in the NCBI database under the GenBank accession number MZ893465.

## 5. Conclusions

Herein, we reported a hypervirulent multidrug-resistant ST13 *K. pneumoniae* strain producing KPC-70, a KPC-3 variant conferring resistance to ceftazidime/avibactam combination. To the best of our knowledge, this is the first report of a CZA-resistant strain in Portugal and the second KPC-70 variant description worldwide. The application of genomic characterization and molecular surveillance promotes the understanding of *K. pneumoniae* population structure, AMR, pathogenicity, and transmission in clinical environments and should be widely encouraged. Further studies are needed to elucidate the molecular features involved in CZA resistance development and spread.

## Figures and Tables

**Table 1:** Antimicrobial susceptibility profile from the ceftazidime/avibactam-resistant *K. pneumoniae* strain.

Antibiotic Tested	AST Dose (μg)	AST ^a^		MIC (mg/L)
		Inhibition Zone (mm)	Interpretation	
Penicillins:				
Amoxicillin/clavulanic acid	20/10	12	R	-
Cephalosporins:				
Cefoxitin	30	16	R	-
Cefotaxime	5	18	R	-
Ceftazidime	10	6	R	-
Ceftazidime/avibactam	10/4	6	R	>256
Carbapenems:				
Imipenem	10	28	S	-
Ertapenem	10	18	R	-
Doripenem	10	23	I	-
Meropenem	10	21	I	-
Monobactams:				
Aztreonam	30	23	I	-
Fluoroquinolones:				
Ciprofloxacin	5	24	I	-
Aminoglycosides:				
Gentamicin	10	10	R	-
Tetracycline:				
Tigecycline	15	22	S	-

^a^ Following European Committee on Antimicrobial Susceptibility Testing (EUCAST) guidelines. MIC = minimum inhibitory concentration; AST = antimicrobial susceptibility testing; S = susceptible standard dosing regimen; I = susceptible, increased exposure; R = resistant.

**Table 2:** Molecular characterization of the ceftazidime/avibactam-resistant *K. pneumoniae*.

ID	MLST	*bla_*Carb	*bla*_Narrow Spectrum	Aminoglycosides	tmp	sul	Quinolones	fos	OmpK	*wzi*	K_locus	O_locus	Fimbriae	Iron Uptake	ICE*Kp*	Genotoxin	Plasmid
FMUL94	ST13	KPC-70	TEM-1; SHV-1; OXA-9	*aac(6′)-Ib*; *aadA*; *aph(3″)-Ib*; *aph(6)-Id*	*dfrA14*	*sul*2	*gyr*A-87N	*fosA*	*OmpK35* *-70%*	*wzi*40	KL3	O1v2	*fimA-fimK*; *mrkA-mrkJ*	*kfu*, *enterobactin(entA–fes)*, *aerobactin(iutA)*, *salmochelin (iroN–iroE)*, *yersiniabactin(fyuA–ybtX)*	*ybt*17; *ICEKp10*	*colibactin* *(clb-c,2-i,l-q)*	ColRNAI; IncFIA (pBK30683); IncFII (pBK30683)

*bla*: β-lactamase; tmp: trimetoprim; sul: sulfanamides; fos: fosfomycin.

## Data Availability

Not applicable.
